# Comparison of Various Soybean Allergen Levels in Genetically and Non-Genetically Modified Soybeans

**DOI:** 10.3390/foods9040522

**Published:** 2020-04-21

**Authors:** Ayato Matsuo, Kaho Matsushita, Ayano Fukuzumi, Naoki Tokumasu, Erika Yano, Nobuhiro Zaima, Tatsuya Moriyama

**Affiliations:** 1 Department of Applied Biological Chemistry, Graduate School of Agriculture, Kindai University, Nara 631-8505, Japan; ayato.m1223@gmail.com (A.M.); 1511430087w@nara.kindai.ac.jp (K.M.); ayano.fukuzumi.0123@gmail.com (A.F.); naoki.tokumasu.1205@gmail.com (N.T.); pyonchangoods@yahoo.co.jp (E.Y.); zaima@nara.kindai.ac.jp (N.Z.); 2 Agricultural Technology and Innovation Research Institute, Kindai University, Kindai University, Nara 631-8505, Japan

**Keywords:** soybean, allergens, allergenicity, genetically modified, Gly m 7

## Abstract

Several analyses of allergen levels have been reported as part of the safety assessment of genetically modified (GM) soybean; however, few comprehensive analyses have included new allergens. Thus, in this study the levels of eight major soybean allergens, including Gly m 7 (a newly reported soybean allergen), were semi-quantitatively detected in six GM soybeans and six non-GM soybeans using antigen-immobilized ELISA and immunoblotting. We also analyzed the IgE-reactivity to these soybeans through immunoblotting, using sera from three soybean-allergic patients. The results showed that there were no significant differences in the levels of the major soybean allergens in the GM and non-GM soybeans. Moreover, there were no significant differences in the serum IgE-reactive protein profiles of the patients, as analyzed using immunoblotting. These results indicate that, in general, CP4-EPSPS-transfected GM soybeans are not more allergenic than non-GM soybeans.

## 1. Introduction

Food resource problems associated with climate change, environmental destruction, and population growth are of increasing concern. As a means to overcome these concerns, scientists have developed “genetic modification technology”, which alters the properties of agricultural products. Using this technology, genetically modified (GM) crops have been developed that are not only more resistant to herbicides but also contain beneficial traits such as drought tolerance, delayed ripening, bacterial disease resistance, high oleic acid levels, and pest resistance, to prepare for an increase in global demand [1,2,3,4]. The total area of cultivation of GM crops has increased worldwide, to 189,800,000 ha in 2017, with four major GM crops: soybeans (50%), corn (31%), cotton (13%), and rapeseed (5%) [5]. Soybeans are not only used as a raw material for soybean oil but are also widely used as foods such as tofu, fermented soybeans (natto), miso, soy sauce, and soy milk, and as additives in various processed foods in the form of soy protein isolate (SPI).

However, ingestion of soybeans can cause allergic reactions, and various soybean allergens have been identified to date [6]. Soybean allergies can be divided into class 1 food allergies and class 2 food allergies based on differences in sensitization routes [7,8]; 7S globulin (Gly m 5) [9,10], 11S globulin (Gly m 6) [11], Gly m 7 [12], Gly m Bd 30K [13,14], Kunitz-type trypsin inhibitor [15], oleosin [16], etc. have been identified as class 1 food allergens causing class 1 allergy. It has been reported that these allergens mainly cause systemic symptoms such as urticaria, diarrhea, vomiting, atopy, and anaphylaxis. Gly m 3 (profilin) [17,18] and Gly m 4 (starvation-associated message 22: SAM22) [18,19,20,21] from soybeans have been reported as class 2 allergens causing class 2 allergy (i.e., pollen–food allergy syndrome [PFAS]). Both Gly m 3 and Gly m 4 are homologues of Bet v 2 and Bet v 1, which are birch pollen allergens that mainly cause oral allergy syndromes (OASs) [18], although severe cases of anaphylaxis with facial swelling, airway narrowing, and breathing difficulties have also been reported [8].

Analyses of variation in the relative levels of known endogenous allergens is required to verify whether genetic transformation or transgenes adversely affect human health and whether the level of endogenous allergens is altered by genetic modification. Therefore, studies using sera from soybean allergy patients with IgE antibodies have been conducted using GM soybeans and non-GM soybeans. Lua et al. conducted an IgE-immunoblot and IgE-ELISA using GM soybeans and a closely related variety of non-GM soybeans; they found that GM soybeans had similar allergenicity to non-GM soybeans and identified no changes in the immunoblot results attributable to genetic modification [2]. Kim et al. reported that IgE-inhibition ELISA using patient serum showed equivalent inhibition in both non-GM soybean and GM soybean extracts, with IgE-immunoblots detecting the most 33 kDa bands in 50% (7/14) of the sera tested and in lanes applied with GM soybean extract and non-GM soybean extract, which were identical to P34 proteins (Gly m Bd 30K). They also argued that the allergenic risk of GM and non-GM soybeans is the same as the allergenic risk of wild-type soybeans because no specific IgE antibodies were detected against the recombinant protein that was genetically integrated into the soybeans, EPSPS (5-*enol*pyruvylshikimate-3-phosphate synthase) [22], which confers resistance to the herbicide glyphosate [23,24]. Tsai et al. also reported no significant differences in Gly m 4 levels in non-GM soybean cultivars and GM soybeans (transfected with EPSPS genes and CaMV 35S promoters) [25]. In addition, there are several reports describing the allergen levels of GM crops [26,27,28,29,30,31]. Thus, while some studies have explored the allergenicity of GM soybeans, there have been no studies focusing on a wide variety of soybean allergen components, and none that explore differential allergenicity of Gly m 7, a recently discovered soybean allergen. Therefore, in this study, to update the allergenicity assessment of GM soybeans, we analyzed the variability of various soybean class 1 food allergens and soybean class 2 (pollinosis-related) food allergen levels in GM soybeans and non-GM soybeans in vitro and compared the patterns of IgE-binding proteins using sera from soybean allergenic patients.

## 2. Materials and Methods

### 2.1. Materials

Horseradish peroxidase (HRP)-labeled anti-rabbit and anti-mouse IgGs were obtained from Thermo Fisher Scientific (Waltham, MA, USA), and HRP-labeled anti-guinea pig IgG was obtained from Jackson ImmunoResearch (West Grove, PA, USA). HRP-labeled anti-human IgE was obtained from Kirkegaard and Perry Laboratories, Inc. (Gaithersberg, MD, USA). ECL^TM^ Western blotting reagent and Hyperfilm^TM^-MP X-ray films were obtained from GE Healthcare (Piscataway, NJ, USA). PVDF membrane (Immobilon^TM^-P) was obtained from Millipore (Billerica, MA, USA).

### 2.2. Soybean Sample Extraction

GM soybeans and non-GM soybeans (controls) were obtained from an anonymous seed company. Each sample (approximately 2.5 g) was mixed with distilled water (25 mL), soaked at room temperature (25 °C) for 4 h, and crushed for 30 s in a mixer. Thereafter, the mixture was squeezed with quadruple gauze to obtain a protein extract. The extract was diluted 20- and 800-fold with distilled water for detection of the allergen levels.

### 2.3. Immunochromatography

To confirm genetic modification of the GM soybean samples, the transgene CP4-EPSPS (EPSPS derived from Agrobacterium CP4 strain) was detected using the Reveal for CP4 Strip Test Kit (Neogen) according to the instruction manual.

### 2.4. Electrophoresis and Immunoblotting

The extracted soybean proteins were subjected to SDS–PAGE. Proteins in the 12.5% gel were stained with Coomassie brilliant blue (CBB) (CBB R-350, GE Healthcare) to visualize the total protein patterns. The immunoblotting analysis was conducted by transferring the SDS–PAGE gel to an Immobilon-P^TM^ PVDF membrane (Millipore) using a semi-dry blotting method [32]. The membrane was incubated in 10 mM PBS (pH = 7.5) containing 0.1% Tween-20 (PBST) and 5% skim milk for blocking. The membrane was then incubated for 1 h at room temperature (25 °C) in a blocking buffer containing allergen-specific antibodies. After the membranes were washed four times with PBST for 10 min, the bound primary antibodies were detected by using HRP-conjugated goat anti-rabbit, anti-mouse, or anti-guinea pig IgG and an ECL^TM^ Western blotting kit (GE Healthcare). The resultant chemiluminescent signals were detected on X-ray film (Hyperfilm^TM^ MP, GE Healthcare). Immunoblotting experiments were performed three times, and band densities were determined using Alpha Ease^TM^ software (Alpha Innotech, San Leandro, CA, USA). The immunoblot results were expressed relative to the value of the control No. 1 sample (C1).

### 2.5. Antibodies Against the Major Soybean Allergens

Mouse monoclonal antibody against Gly m Bd 30 K [6] was kindly provided by Dr. Tadashi Ogawa (Professor Emeritus at Kyoto University). The rabbit polyclonal antibodies against Gly m 5 (7S globulin; β-conglycinin; αʹ, α, and β subunits) were obtained as previously described [33]. The mouse polyclonal antibodies against Gly m 6 (11S globulin; glycinin) were obtained by immunizing mice with purified 11S globulin in our laboratory. The rabbit polyclonal antibodies against Gly m 4 were obtained by immunizing rabbit with recombinant Gly m 4 in our laboratory. The rabbit polyclonal antibodies against oleosin were also obtained by immunizing rabbit with purified oleosin in our laboratory. The guinea pig polyclonal antibodies against Gly m 3 were also obtained by immunizing guinea pigs with recombinant Gly m 3 in our laboratory [34]. The rabbit antibody against soybean trypsin inhibitor was obtained from Rockland (Gilbertsville, PA, USA). Gly m 7 is a seed biotinylated protein with a single binding site for biotin. Two methods were used to detect Gly m 7: rabbit-derived peptide antibodies (unpublished data) that can detect the peptide moiety of Gly m 7, and HRP-labeled streptavidin that binds specifically to biotin. The specific reactivities of these antibodies have been confirmed in our previous studies. The soybean allergens detected in this study are listed in Table 1.

### 2.6. ELISA Using Allergen-Specific Antibodies

ELISA was used to evaluate the allergen levels of non-GM (control) and GM soybeans. ELISA plates were coated with sequentially diluted (100- to 1,000,000-fold diluted) soybean extracts. After sample coating, plates were blocked with Blocking one (nacarai tesque, Kyoto, Japan, dilution 1:5) for 1 h at room temperature (25 °C) and then washed with PBST three times. Next, diluted allergen-specific antibodies were added to the wells, and samples were incubated for 1 h at 37 °C. Plates were then washed with PBST five times and HRP-labeled secondary antibodies were added to the wells. Plates were then incubated for 1 h at 37 °C and then washed with PBST five times. The bound HRP-labeled secondary antibodies were visualized by reaction with 100 μL of tetramethylbenzidine (TMB) peroxidase substrate (KPL, Gaithersburg, MD, USA) for 5–15 min. The reaction was stopped by adding 100 μL of 1 M phosphoric acid to provide a stable endpoint color. The absorption was measured at 450 nm using an ARVOsx-1 1420 multilabel counter (PerkinElmer Life Sciences, Boston, MA, USA). Measurements were performed three times, and the mean absorbance values were calculated.

### 2.7. Detection of IgE-Binding Proteins using Patient Sera

A total of 3 commercially available soybean allergy patient sera were purchased from Kokusai Bio Co., ltd (Tokyo, Japan). The patients were all soybean class 1 food allergy patients from the United States. Immunoblotting and ELISA were performed using patient sera. HRP-labeled anti-human IgE antibody was used as a secondary antibody. The detected IgE-binding protein bands were exposed to X-ray films and captured by a scanner; band densities were determined using Alpha Ease^TM^ software (Alpha Innotech, San Leandro, CA, USA).

### 2.8. Statistical Analysis

Results are expressed as the mean ± standard deviation (SD). Data was analyzed by Student’s t-test with Excel Statistics software (SSRI Co., Tokyo, Japan). *P* value < 0.05 was considered statistically significant.

## 3. Results

### 3.1. Confirmation of Genetically Modified and Non-Genetically Modified Soybeans

**Figure 1:**
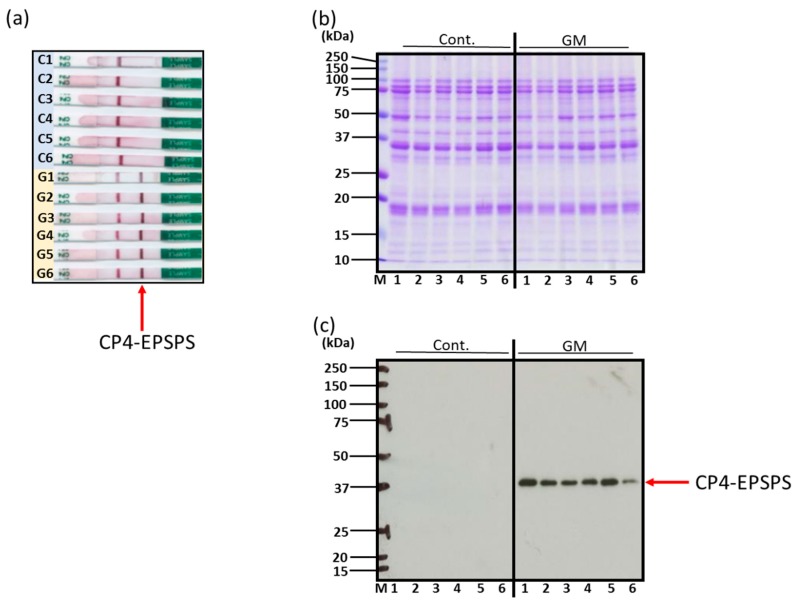
Characterization and confirmation of genetically modified (GM) and non-genetically modified (non-GM) soybeans. Indicated soybeans were extracted and subjected to immunochromatography (**a**), SDS–PAGE followed by CBB (CBB R-350, GE Healthcare) staining (**b**), and immunoblotting for detection of recombinant protein CP4-EPSPS (**c**). (C1–C6), non-GM soybeans; (G1–G6), GM soybeans.

Proteins were extracted from 12 kinds of soybeans including six kinds of GM soybeans and six kinds of non-GM soybeans. Detailed information about each GM and non-GM soybean cultivar was not available for blind testing. However, these soybeans were all popular cultivars in the world. Immunochromatographic analysis of soybean extracts detected CP4-EPSPS in all six kinds of GM soybeans, confirming that the samples were GM soybeans, and did not detect CP4-EPSPS in all six kinds of non-GM soybeans (Figure 1a). SDS–PAGE of protein extracts from GM soybeans and non-GM soybeans as visualized by CBB showed no obvious differences (Figure 1b). Immunoblotting of the soybean protein extracts using antibodies against CP4-EPSPS, the recombinant gene product, detected CP4-EPSPS in the six GM soybean species but not in the six non-GM soybean species (Figure 1c).

### 3.2. Comparative Levels of Pollinosis-Related Soybean Allergens (Gly m 4 and Gly m 3) in GM-Soybean and Non-GM Soybean Extracts

**Figure 2:**
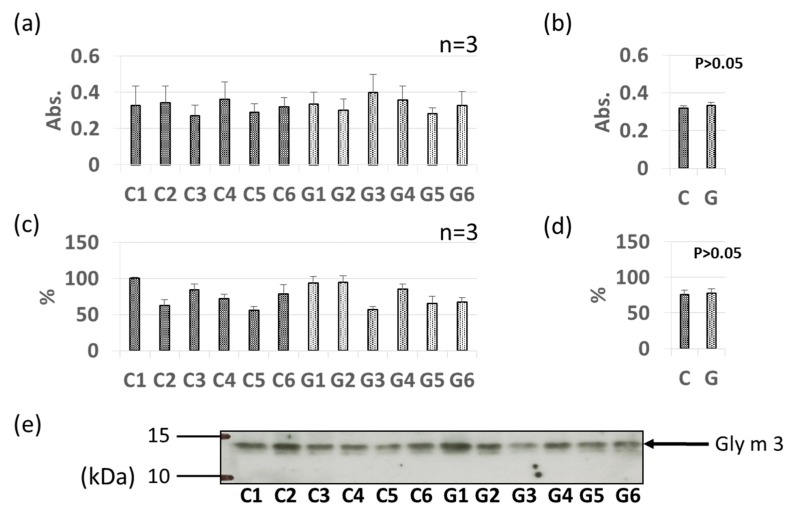
Comparison of Gly m 3 levels in GM-and non-GM soybeans by ELISA (**a**,**b**) and immunoblotting (**c**–**e**) using guinea pig-derived polyclonal antibodies. Soybean protein extracts were evaluated by ELISA (**a**,**b**) and immunoblotting (**c**–**e**) for detection of Gly m 3 levels. The ELISA data are presented in absorbance values (Abs). The individual data from six GM-and non-GM soybeans (**a**,**c**) are presented as the mean ± SD of three independent replicates. The collated data (**b**,**d**) are presented as the mean ± SD of all individual data points from the control (C1–C6, six non-GM soybeans) or experimental (G1–G6, six GM soybeans) groups relative to the value of control number 1 (C1). A representative immunoblot is also provided (**e**).

**Figure 3:**
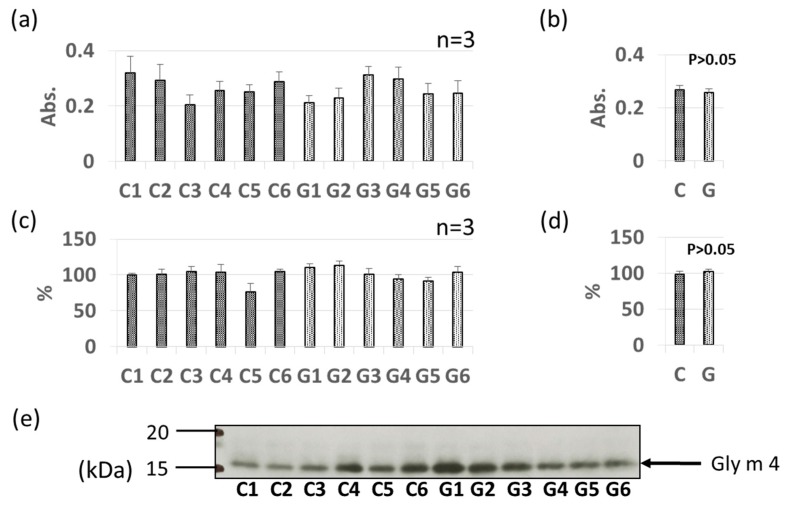
Comparison of Gly m 4 levels in GM-and non-GM soybeans by ELISA (**a**,**b**) and immunoblotting (**c**–**e**) using rabbit-derived polyclonal antibodies. Soybean protein extracts were evaluated by ELISA (**a**,**b**) and immunoblotting (**c**–**e**) for detection of Gly m 4 levels. The ELISA data are presented in absorbance values (Abs). The individual data from six GM-and non-GM soybeans (**a**,**c**) are presented as the mean ± SD of three independent replicates. The collated data (**b**,**d**) are presented as the mean ± SD of all individual data points from the control (C1–C6, six non-GM soybeans) or experimental (G1–G6, six GM soybeans) groups relative to the value of control number 1 (C1). A representative immunoblot is also provided (**e**).

Pollinosis-related soybean allergens Gly m 3 and Gly m 4 were detected by ELISA and immunoblotting in soybean extracts, as shown in Figure 2 and Figure 3. Allergen content varied by individual sample; however, no significant difference in allergen content was found between the non-GM soybean and GM soybean groups for Gly m 3 or Gly m 4 by ELISA (Figure 2a,b, Figure 3a,b). Immunoblotting analysis revealed unique bands at approximately 13 kDa and 17 kDa identifying Gly m 3 (Figure 2c,e) and Gly m 4 (Figure 3c,e) in soybean extracts, respectively. No significant difference in allergen content was found between the non-GM soybean and GM soybean groups for Gly m 3 or Gly m 4 by immunoblotting (Figure 2d and Figure 3d).

### 3.3. Comparative Levels of Other Soybean Allergens in GM-Soybean and non-GM Soybean

**Figure 4:**
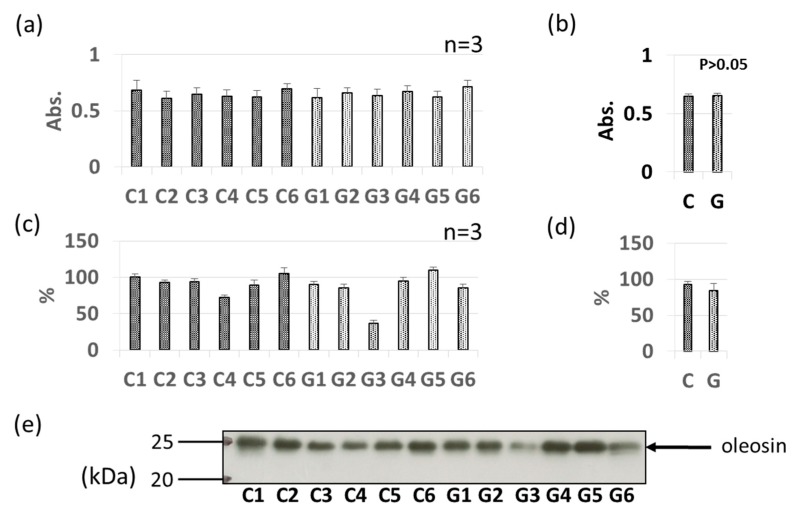
Comparison of oleosin levels in GM-and non-GM soybeans by ELISA (**a**,**b**) and immunoblotting (**c**–**e**) using rabbit-derived polyclonal antibodies. Soybean protein extracts were evaluated by ELISA (**a**,**b**) and immunoblotting (**c**–**e**) for detection of oleosin levels. The ELISA data are presented in absorbance values (Abs). The individual data from six GM-and non-GM soybeans (**a**,**c**) are presented as the mean ± SD of three independent replicates. The collated data (**b**,**d**) are presented as the mean ± SD of all individual data points from the control (C1–C6, six non-GM soybeans) or experimental (G1–G6, six GM soybeans) groups relative to the value of control number 1 (C1). A representative immunoblot is also provided (**e**).

**Figure 5:**
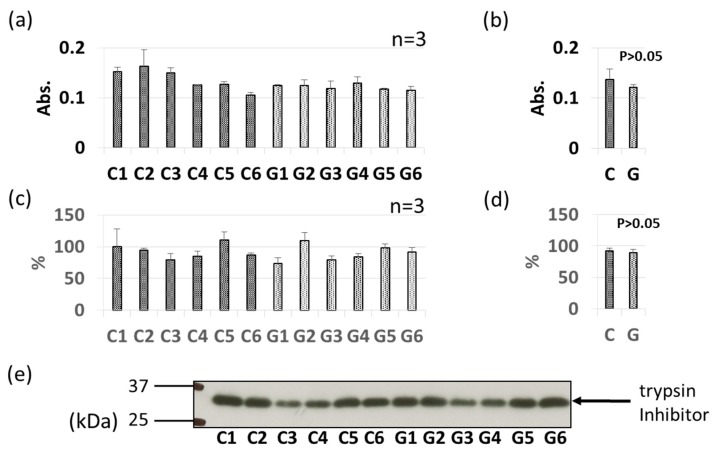
Comparison of trypsin inhibitor levels in GM-and non-GM soybeans by ELISA (**a**,**b**) and immunoblotting (**c**–**e**) using a rabbit-derived antibody. Soybean protein extracts were evaluated by ELISA (**a**,**b**) and immunoblotting (**c**–**e**) for detection of trypsin inhibitor levels. The ELISA data are presented in absorbance values (Abs). The individual data from six GM-and non-GM soybeans (**a**,**c**) are presented as the mean ± SD of three independent replicates. The collated data (**b**,**d**) are presented as the mean ± SD of all individual data points from the control (C1–C6, six non-GM soybeans) or experimental (G1–G6, six GM soybeans) groups relative to the value of control number 1 (C1). A representative immunoblot is also provided (**e**).

**Figure 6:**
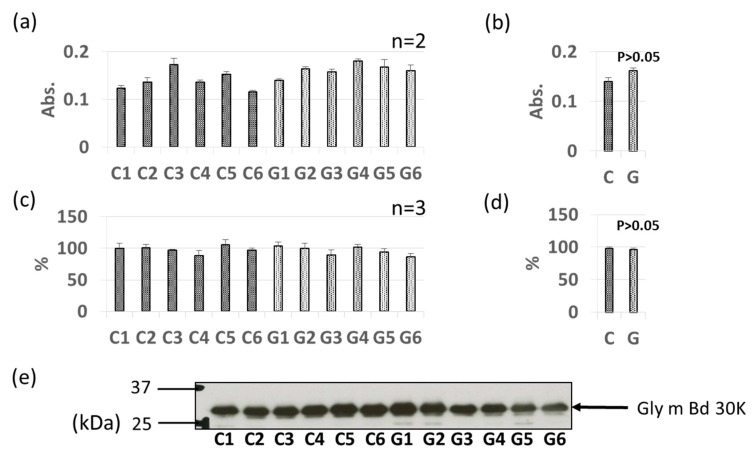
Comparison of Gly m Bd 30K levels in GM-and non-GM soybeans by ELISA (**a**,**b**) and immunoblotting (**c**–**e**) using a mouse-derived monoclonal antibody. Soybean protein extracts were evaluated by ELISA (**a**,**b**) and immunoblotting (**c**–**e**) for detection of Gly m Bd 30K levels. The ELISA data are presented in absorbance values (Abs). The individual data from six GM-and non-GM soybeans (**a**,**c**) are presented as the mean ± SD of three independent replicates. The collated data (**b**,**d**) are presented as the mean ± SD of all individual data points from the control (C1–C6, six non-GM soybeans) or experimental (G1–G6, six GM soybeans) groups relative to the value of control number 1 (C1). A representative immunoblot is also provided (**e**).

**Figure 7:**
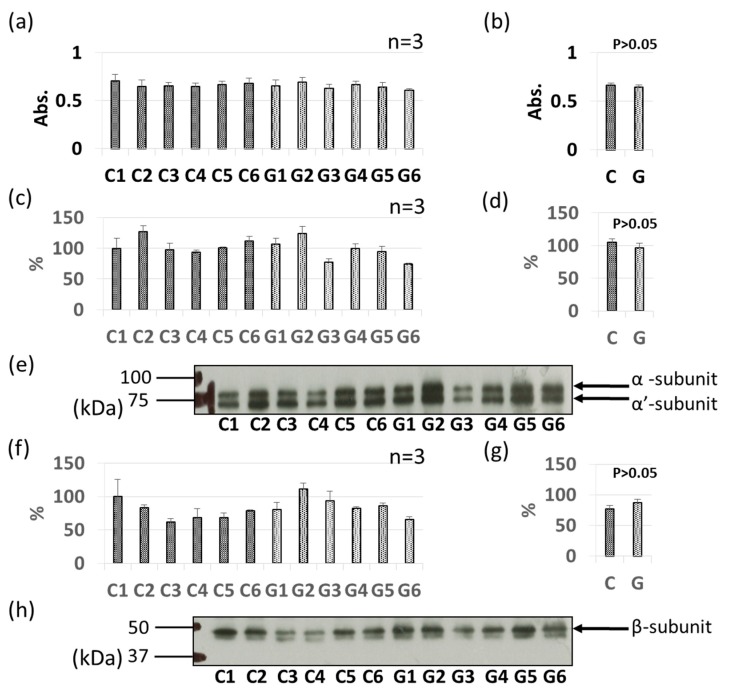
Comparison of Gly m 5 levels in GM-and non-GM soybeans by ELISA (**a**,**b**) and immunoblotting (**c–h**) using rabbit-derived polyclonal antibodies**.** Soybean protein extracts were evaluated by ELISA (**a**,**b**) and immunoblotting (**c**–**h**) for detection of Gly m 5 levels. The ELISA data are presented in absorbance values (Abs). The α- and α’-subunits of Gly m 5 were detected (**c**–**e**) separately from the β-subunit of Gly m 5 (**f**–**h**). The individual data from six GM-and non-GM soybeans (**a**–**f**) are presented as the mean ± SD of three independent replicates. The collated data (**b**,**d**,**g**) are presented as the mean ± SD of all individual data points from the control (C1–C6, six non-GM soybeans) or experimental (G1–G6, six GM soybeans) groups relative to the value of control number 1 (C1). Representative immunoblots are also provided (**e**,**h**).

**Figure 8:**
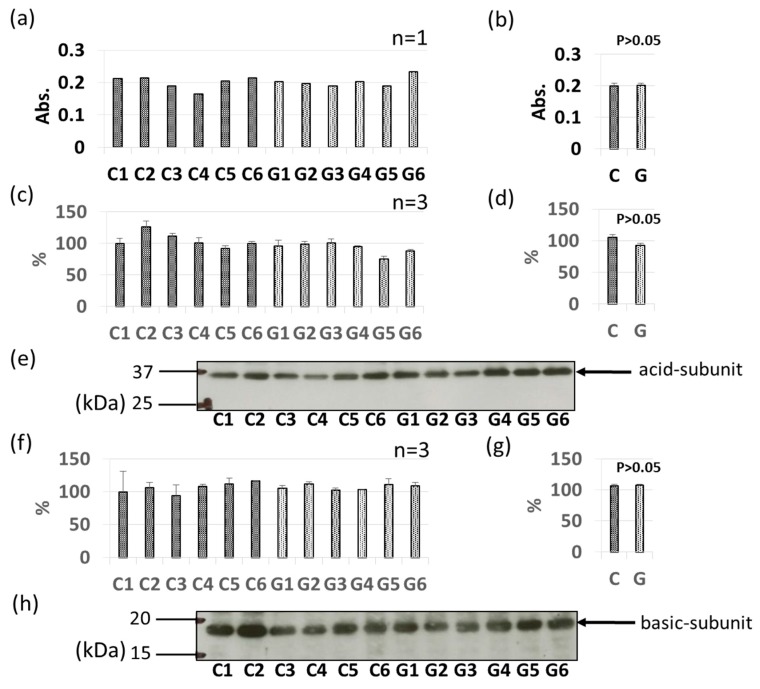
Comparison of Gly m 6 levels in GM-and non-GM soybeans by ELISA (**a**,**b**) and immunoblotting (**c**–**h**) using mouse-derived polyclonal antibodies. Soybean protein extracts were evaluated by ELISA (**a**,**b**) and immunoblotting (**c**–**h**) for detection of Gly m 6 levels. The ELISA data are presented in absorbance values (Abs). The acidic subunit of Gly m 6 was detected (**c**–**e**) separately from the basic subunit of Gly m 6 (**f**–**h**). The individual data from six GM-and non-GM soybeans (**c**,**f**) are presented as the mean ± SD of three independent replicates. The collated data (**b**,**d**,**g**) are presented as the mean ± SD of all individual data points from the control (C1–C6, six non-GM soybeans) or experimental (G1–G6, six GM soybeans) groups relative to the value of control number 1 (C1). Representative immunoblots are also provided (**e**,**h**).

**Figure 9:**
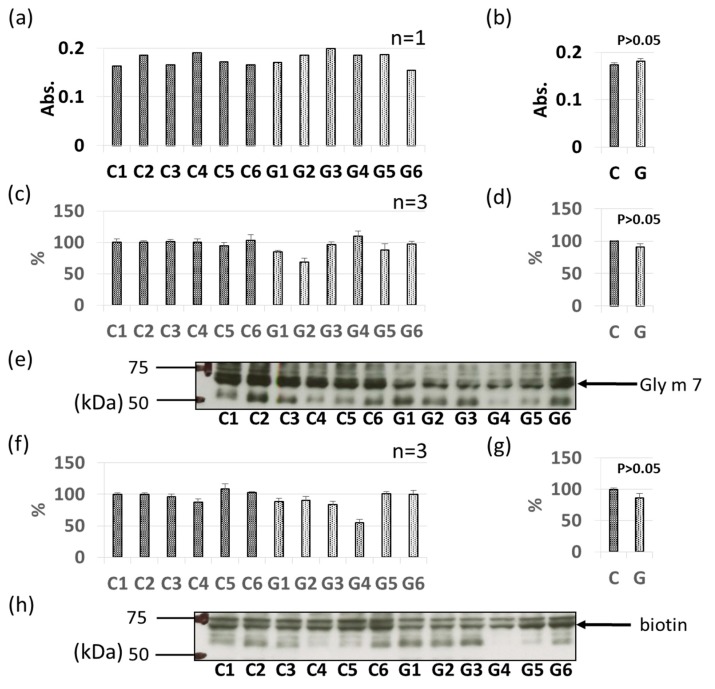
Comparison of Gly m 7 levels in GM-and non-GM soybeans by ELISA (**a**,**b**) and immunoblotting (**c**–**h**). Soybean protein extracts were evaluated by ELISA (**a**,**b**) and immunoblotting (**c**–**h**) for detection of Gly m 7 levels. The ELISA data are presented in absorbance values (Abs). Immunoblotting for Gly m 7 levels was performed using a rabbit-derived peptide-antibody (**c**–**e**) and streptavidin–HRP for the biotin moiety of Gly m 7 (**f**–**h**). The individual data from six GM-and non-GM soybeans (**c**,**f**) are presented as the mean ± SD of three independent replicates. The collated data (**b**,**d**,**g**) are presented as the mean ± SD of all individual data points from the control (C1–C6, six non-GM soybeans) or experimental (G1–G6, six GM soybeans) groups relative to the value of control number 1 (C1). Representative detections are also provided (**e**,**h**). ELISA and immunoblotting were performed using animal-derived antibodies or streptavidin–HRP as described in Section 2.5.

As with Gly m 3 and Gly m 4, other soybean allergen levels in control (non-GM soybeans) and GM soybeans were detected and compared relatively based on ELISA and immunoblotting (Figure 4, Figure 5, Figure 6, Figure 7, Figure 8 and Figure 9). There were no significant differences in levels of oleosin between the control (non-GM soybeans) and GM soybean groups (Figure 4) as determined by ELISA and immunoblotting. Trypsin inhibitor (Figure 5a–e) and Gly m Bd 30 K (Figure 6a–e) levels were also similar between the groups, with no significant differences between the non-GM soybean and GM soybean groups. The major storage protein, Gly m 5 (7S globulins), is composed of three subunits: α, α’, and β subunit. ELISA results showed no significant differences in the banding intensity of Gly m 5 between the control and experimental group (Figure 7a,b) and no significant differences in the banding intensities of each of the three subunits (α + α’, and β subunit) by immunoblotting (Figure 7c–f). Additionally, the major storage protein Gly m 6 (11S globulin) is composed of an acidic subunit (AS) and basic subunit (BS). There were no significant differences in the levels of Gly m 6 between the control non-GM soybean and the GM soybean groups as determined by ELISA and immunoblotting (Figure 8). Levels of Gly m 7, a recently discovered soybean allergen, were also examined; there were no significant differences in Gly m 7 levels in the non-GM soybean and the GM soybean groups as determined by ELISA (Figure 9a,b) or by immunoblotting using two different anti-Gly m 7 antibodies (Figure 9c–f).

### 3.4. IgE-ELISA and IgE-Immunoblotting using Patient Serum

**Figure 10:**
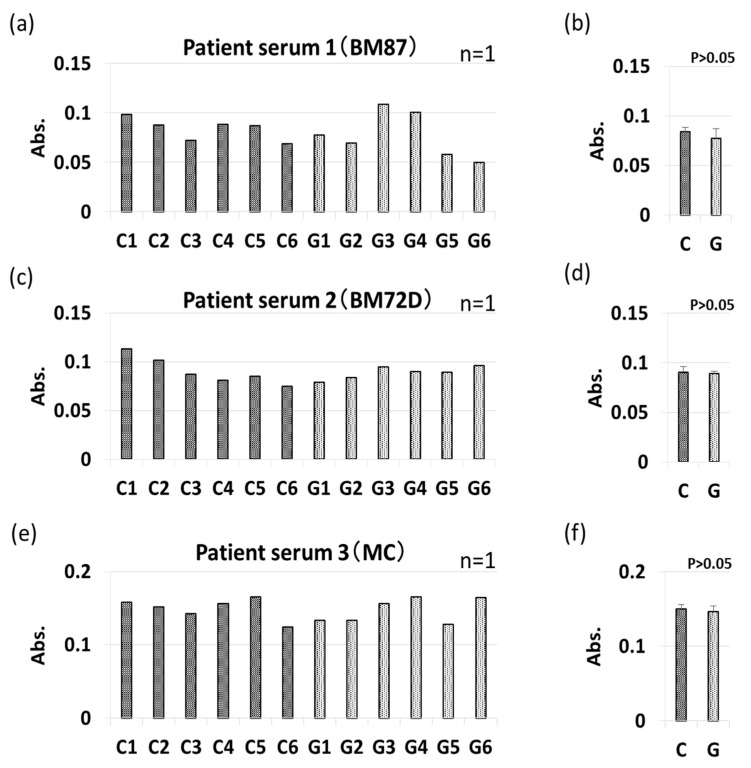
IgE-ELISA of GM-and non-GM soybeans using patient sera. IgE-ELISA performed using sera from three soybean-allergenic patients is shown as follows: patient serum 1 (**a**,**b**), patient serum 2 (**c**,**d**), patient serum 3 (**e**,**f**). The collated data (**b**,**d**,**f**) are presented as the mean ± SD of all individual data points from the control (C1–C6, non-GM soybeans) or experimental (G1–G6, GM soybeans) groups for each serum sample.

**Figure 11:**
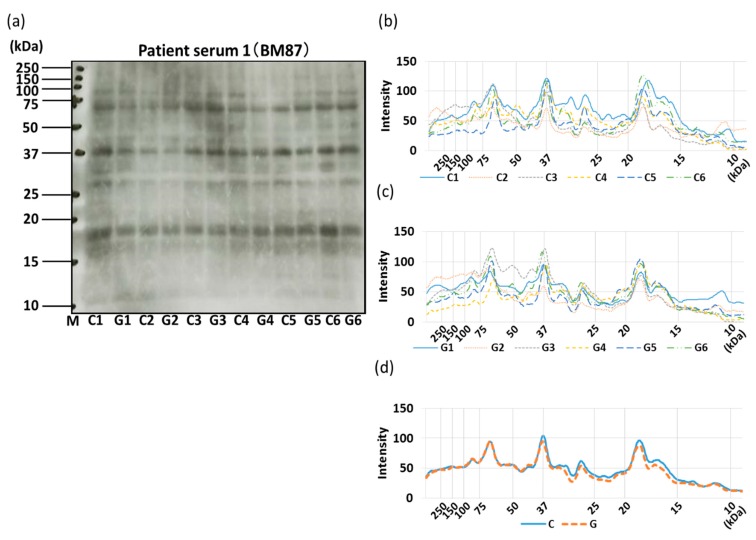
IgE-immunoblotting of GM-and non-GM soybeans using patient serum 1. A representative IgE-immunoblot of GM soybeans (G1–G6) and non-GM soybeans (C1–C6) is shown (**a**). Immunoblots were analyzed on each soybean sample once by densitometry and separated into non-GM soybean (**b**) and GM soybean groups (**c**). The average IgE-immunoblot profiles of non-GM soybeans and GM soybeans were calculated (**d**).

**Figure 12:**
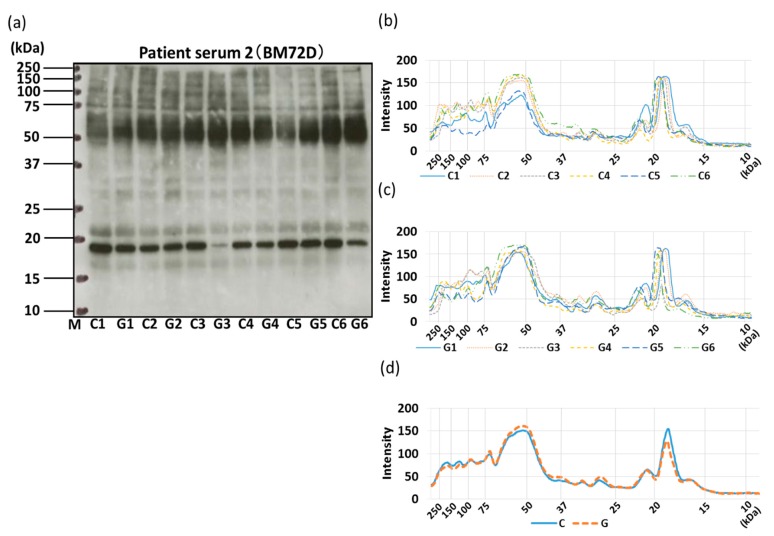
IgE-immunoblotting of GM-and non-GM soybeans using patient serum 2. A representative IgE-immunoblot of GM soybeans (G1–G6) and non-GM soybeans (C1–C6) is shown (**a**). Immunoblots were analyzed on each soybean sample once by densitometry and separated into non-GM soybean (**b**) and GM soybean groups (**c**). The average IgE-immunoblot profiles of non-GM soybeans and GM soybeans were calculated (**d**).

**Figure 13:**
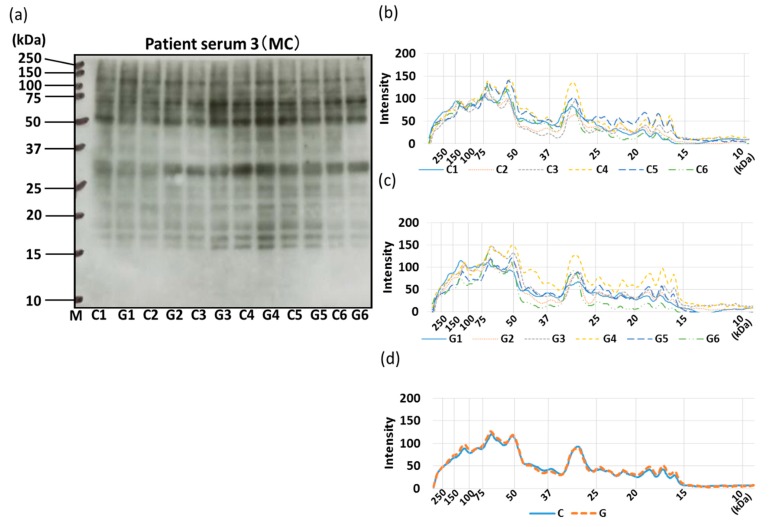
IgE-immunoblotting of GM-and non-GM soybeans using patient serum 3. A representative IgE-immunoblot of GM soybeans (G1–G6) and non-GM soybeans (C1–C6) is shown (**a**). Immunoblots were analyzed on each soybean sample once by densitometry and separated into non-GM soybean (**b**) and GM soybean groups (**c**). The average IgE-immunoblot profiles of non-GM soybeans and GM soybeans were calculated (**d**).

The allergenicity of non-GM soybeans and GM soybeans was then compared by IgE-ELISA and immunoblotting using the sera of three commercial soybean-allergic patients (Figure 10, Figure 11, Figure 12 and Figure 13). Soybean strains exhibited varying levels of allergenicity to serum IgE as determined by IgE-ELISA, but there was no significant difference between the allergenicity of the non-GM soybean and the GM soybean groups in the sera of all three patients (Figure 10). Furthermore, IgE-binding patterns were evaluated by IgE-immunoblotting and analyzed both visually and by densitometric analysis. IgE-immunoblotting revealed qualitative differences in IgE-binding patterns for different soybean strains in the sera of different patients. For example, in patient serum 1, the peak densitometric intensities of IgE-bound proteins were found at approximately 72 kDa, 37 kDa, and 18 kDa (Figure 11). In patient serum 2, the peak densitometric intensities of IgE-bound proteins were found at approximately 50 kDa and 18 kDa (Figure 12). In patient serum 3, the peak densitometric intensities of IgE-bound proteins were found at approximately 75–50 kDa and 30 kDa (Figure 13). The peak densitometric intensities of IgE-bound proteins should supposedly correlated with the molecular weight of major soybean allergens, such as Gly m 5 and Gly m 6 (full list in Table 1). There were no qualitative differences in the IgE-immunoblotting results of the non-GM soybean and the GM soybean groups for all three patients’ sera; specifically, IgE-binding bands were not increased or decreased in GM soybeans (Figure 11, Figure 12 and Figure 13). These results indicate that the patient-serum IgE does not specifically bind to the transgene product (CP4-EPSPS).

## 4. Discussion

In immunochromatography, two lines specific to genetic recombination were detected from six GM soybeans. Twelve soybean strains (six non-GM and six GM strains) were evaluated to determine whether genetic modification affected expression of previously identified allergens or IgE allergenicity. Immunoblotting using antibodies to detect the recombinant gene product CP4-EPSPS revealed expression of EPSPS in the six GM soybean strains but not in the non-GM strains. These results confirmed that all six GM soybean species used in this study had been genetically modified and demonstrated that all six non-GM soybean species had not been genetically modified to express EPSPS (Figure 1a–c). SDS–PAGE and CBB staining showed no visible differences in the protein expression profiles of GM soybean and non-GM soybean groups as a whole; and it was speculated that no new protein bands detectable at the CBB staining levels were found to be generated, increased, decreased, or eliminated by the introduction of CP4-EPSPS.

One of the major storage proteins, Gly m 5 (7S globulin: β-conglycinin), consists of three subunits (α subunit, approximately 68 kDa; α’-subunit, approximately 72 kDa; β-subunit, approximately 50 kDa); the α subunit was first identified as an allergen, and subsequent studies using IgE antibodies from the sera of soybean-allergic patients revealed that the α’and β subunits were also allergens [35]. Structural homology between these three subunits is relatively high. Gly m 5, which is found in tofu, a processed soybean food, is stable against pepsin-digestion, and has been reported to be responsible for food-dependent exercise-induced anaphylaxis (FDEIA) [36]. Gly m 6 (11S globulin) is also known to be a major soybean allergen [11]. Both Gly m 5 and Gly m 6 are seed storage proteins that account for about 70% of all seed proteins [37]. In this study, we found that the allergen levels of these two major seed storage proteins do not differ significantly between GM soybeans and non-GM soybeans.

The newly discovered soybean allergen Gly m 7 is a unique seed-specific biotinylated protein (SBP) that belongs to the late embryogenesis (LEA) protein family. It was discovered by Riascos et al. in a study evaluating the allergenicity of boiled lentils. The authors generated full-length cDNA clones encoding SBPs identified in lentils from developing soybean seeds and successfully expressed the protein as His-tagged recombinant proteins (rSBP) in *Escherichia coli*. They succeeded in purification of naturally-derived soybean SBP (nSBP-soy, later named Gly m 7) and confirmed IgE-positive and basophil-stimulating effects between soybean and peanut-allergic sera, suggesting that Gly m 7 may cause IgE-mediated allergic reactions [12]. In the present study, we detected and compared the levels of this novel allergen by two methods (peptide-antibody and biotin-detection) and found no significant differences between its expression levels in the GM soybean and non-GM soybean groups in either case.

Gly m Bd 30K is the predominant allergen found in soybeans. It is a 32 kDa protein also known as the vacuolar protein p34 in soybeans. It has been identified as an oil-body associated component of soybean seeds [13]. A Kunitz-type trypsin inhibitor was identified as a soybean allergen in 1980 [15] and was reported to be an occupational inhalant allergen [38]. There were no significant differences in protein levels between the GM soybean and non-GM soybean groups for any of these classical allergens.

The soybean allergen Gly m 4, which belongs to the pathogenesis-related protein10 (PR-10) family, is a homolog of the pollen-antigen Bet v 1 of birch. Gly m 4 has been widely reported to cross-react with food PR-10 proteins. Berkner et al. reported immunoblot inhibition assays using recombinant (r)Gly m 4 indicating that rBet v 1 was most inhibited by IgE-binding to rGly m 4 (100%), followed by rGly m 4, apple (rMal d 1), and cherry (rPru av 1) [21]. Gly m 3 (profilin) also cross-reacts with Bet v 2, another birch pollen-antigen, and is an actin-binding protein present in all organisms (including plants and animals), with more than 70% homology between Gly m 3 and Bet v 2. Rihs et al. reported that there were common IgE-binding epitopes in rGly m 3 and rBet v 2 as determined by EAST (enzyme allergosorbent test) inhibition assays using sera from non-soybean-allergic patients with cypress pollinosis; furthermore, preincubation of sera with rGly m 3 completely inhibited IgE binding to rBet v 2 [17]. Quantification of Gly m 3 in some soy products by indirect ELISA was also reported [39].

The immunoblotting and ELISA assays in this study indicated that the levels of these two pollinosis-related soybean allergens (Gly m 3 and Gly m 4) were not increased or decreased by genetic modification (Figure 2 and Figure 3). Interestingly, the levels of these two allergens have been reported to be significantly increased by worm wounding [28]. In particular, since Gly m 4 is a pathogenesis-related protein, it is known that its expression is induced by stresses such as disease. Therefore, it is suggested that the level of these allergens is greatly affected by the cultivation environment. Our results suggest that the level of PR protein is unlikely to be increased by the genetic modification process.

Next, IgE-binding was evaluated using the serum of three soybean-allergic patients in order to evaluate the allergenic capacity of GM and non-GM soybeans from a clinical perspective. The IgE-ELISA results showed no significant differences between the GM soybean and the non-GM soybean groups when testing the sera of all three patients (Figure 10). IgE-immunoblotting revealed qualitative differences in IgE-binding patterns for different soybean strains in the sera of different patients. These IgE-binding proteins were supposed to be soybean-major allergens such as Gly m 5, Gly m 6 (Figure 11a–c, Figure 12a–c, Figure 13a–c). There were no qualitative differences in the IgE-immunoblotting results of the non-GM soybean and the GM soybean groups for all three patients’ sera, indicating that the allergen-candidate molecules did not differ between GM soybeans and non-GM soybeans (Figure 11d, Figure 12d, Figure 13d). These results also indicate that patient-serum IgE does not specifically bind to the transgene product (CP4-EPSPS).

Taken together, it was concluded that the CP4-EPSPS transfected GM soybeans used in this study had similar allergen abundance levels and allergen reactivity to the non-GM soybeans. These results are similar to other GM soybean allergenicity studies conducted thus far. In this study, we found that GM technology did not increase or decrease the level of endogenous soybean allergen proteins, nor did it induce the appearance of new soybean allergens. However, further investigation of the allergenicity of GM soybeans will be necessary for more rigorous evaluation. Research on more soybean varieties, changes in allergenicity due to changes in the cultivation environment, and sensitization potencies, as well as the allergen levels of GM- and non-GM soybeans should be considered.

## Figures and Tables

**Table 1:** Various soybean allergens detected in this study.

Allergen Name	Molecular Mass (kDa)	Features	References
Soybean food allergens (class 1 food allergens)			
Gly m 5 (7S globulin)	72, 68, 50	Major storage protein, glycoprotein	[9,10]
Gly m 6 (11S globulin)	34, 20	Major storage protein	[11]
Gly m 7	76	Seed-specific biotinylated protein (SBP)	[12]
Gly m Bd 30K (P34)	34 (30)	Homologous to papain	[13,14]
Kunitz-type trypsin inhibitor	18	trypsin inhibitor	[15]
Oleosin	22–24	Oil body associated protein	[16]
Pollen-related soybean allergens (class 2 food allergens)			
Gly m 3	14	Profilin, Bet v 2 homolog	[17,18]
Gly m 4	17	PR-10 family, Bet v 1 homolog	[18,19,20,21]

## References

[1] Chrispeels M.J. (2014). Global production and consumption of genetically engineered crops. J. Huazhong Agric. Univ..

[2] Lua M., Jina Y., Weber B.B., Goodman R.E. (2018). A comparative study of human IgE binding to proteins of a genetically modified (GM) soybean and six non-GM soybeans grown in multiple locations. Food Chem. Toxicol..

[3] Ichim M.C. (2019). The Romanian experience and perspective on the commercial cultivation of genetically modified crops in Europe. Transgenic Res..

[4] Shukla M., Al-Busaidi K.T., Trivedi M., Tiwari R.K. (2018). Status of research, regulations and challenges for genetically modified crops in India. GM Crops Food.

[5] ISAAA (2017). Global Status of Commercialized Biotech/GM Crops: Biotech Crop Adoption Surges as Economic Benefits Accumulate in 22 Years.

[6] Ogawa T., Samoto M., Takahashi K. (2000). Soybean allergens and hypoallergenic soybean products. J. Nutr. Sci. Vitaminol..

[7] Yagami T. (2002). Allergies to Cross-Reactive Plant Proteins. Latex-fruit syndrome is comparable with pollen-food allergy syndrome. Int. Arch. Allergy Immunol..

[8] Amlot P.L., Kemeny D.M., Zachary C., Parkes P., Lessof M.H. (1987). Oral allergy syndrome (OAS): Symptoms of IgE-mediated hypersensitivity to foods. Clin. Allergy.

[9] Ogawa T., Bando N., Tsuji H., Nishikawa K., Kitamura K. (1995). α-Subunit of β-Conglycinin, an allergenic protein recognized by IgE Antibodies of soybean-sensitive patients with atopic dermatitis. Biosci. Biotechnol. Biochem..

[10] Maruyama N., Sato S., Cabanos C., Tanaka A., Ito K., Ebisawa M. (2018). Gly m 5/Gly m 8 fusion component as a potential novel candidate molecule for diagnosing soya bean allergy in Japanese children. Clin. Exp. Allergy.

[11] Holzhauser T., Wackermann O., Ballmer-Weber B.K., Bindslev-Jensen C., Scibilia J., Perono-Garoffo L., Utsumi S., Poulsen L.K., Vieths S. (2009). Soybean (Glycine max) allergy in Europe: Gly m 5 (β-conglycinin) and Gly m 6 (glycinin) are potential diagnostic markers for severe allergic reactions to soy. J. Allergy Clin. Immunol..

[12] Riascos J.J., Weissinger S.M., Weissinger A.K., Kulis M., Burks A.W., Pons L. (2016). The Seed Biotinylated Protein of Soybean (Glycine max): A BoilingResistant New Allergen (Gly m 7) with the Capacity to Induce IgE Mediated Allergic Responses. J. Agric. Food Chem..

[13] Ogawa T., Tsuji H., Bando N., Kitamura K., Zhu Y.L., Hirano H., Nishikawa K. (1993). Identification of the soybean allergenic protein, Gly m Bd 30K, with the soybean seed 34-kDa oil-body-associated protein. Biosci. Biotechnol. Biochem..

[14] Ogawa T., Bando N., Tsuji H., Okajima H., Nishikawa K., Sasaoka K. (1991). Investigation of the IgE-binding protein in soybeans by immunoblotting with the sera of the soybean-sensitive patient with atopic dermatitis. J. Nutr. Sci. Vitaminol..

[15] Moroz L.A., Yang W.H. (1980). Kunitz Soybean Trypsin Inhibitor—A Specific Allergen in Food Anaphylaxis. N. Engl. J. Med..

[16] Cao Y., Zhao L., Ying Y., Kong X., Hua Y., Chen Y. (2015). The characterization of soybean oil body integral oleosin isoforms and the effects of alkaline pH on them. Food Chem..

[17] Rihs H.P., Chen Z., Ruëff F., Petersen A., Rozynek P., Heimann H., Baur X. (1999). IgE binding of the recombinant allergen soybean profilin (rGly m 3) is mediated by conformational epitopes. J. Allergy Clin. Immunol..

[18] Yagami A., Inaba Y., Kuno Y., Suzuki K., Tanaka A., Sjolander S., Saito H., Matsunaga K. (2009). Two cases of pollen-food allergy syndrome to soy milk diagnosed by skin prick test, specific serum immunoglobulin E and microarray analysis. J. Dermatol..

[19] Kleine-Tabbe J., Vogel L., Crowell D.N., Haustein U.F., Vieths S. (2002). Severe oral allergy syndrome and anaphylactic reactions caused by a Bet v 1- related PR-10 protein in soybean, SAM22. J. Allergy Clin. Immunol..

[20] Mittag D., Vieths S., Vogel L., Becker W.M., Rihs H.P., Helbling A., Wüthrich B., Ballmer-Weber B.K. (2004). Soybean allergy in patients allergic to birch pollen: Clinical investigation and molecular characterization of allergens. J. Allergy Clin. Immunol..

[21] Berkner H., Neudecker P., Mittag D., Ballmer-Weber B.K., Schweimer K., Vieths S., Rösch P. (2009). Cross-reactivity of pollen and food allergens: Soybean Gly m 4 is a member of the Bet v 1 superfamily and closely resembles yellow lupine proteins. Biosci. Rep..

[22] Kim S.H., Kim H.M., Ye Y.M., Kim S.H., Nahm D.H., Park H.S., Ryu S.R., Lee B.O. (2006). Evaluating the Allergic Risk of Genetically Modified Soybean. Yonsei Med. J..

[23] Wu H., Wang X., Zhou X., Zhang Y., Huang M., He J., Shen W. (2017). Targeting the middle region of CP4-EPSPS protein for its traceability in highly processed soy-related products. J. Food Sci. Technol..

[24] Funke T., Healy-Fried M.L., Han H., Alberg D.G., Bartlett P.A., Schönbrunn E. (2007). Differential Inhibition of Class I and Class II 5-Enolpyruvylshikimate-3-phosphate Synthases by Tetrahedral Reaction Intermediate Analogues. Biochemistry.

[25] Tsai J.J., Chang C.Y., Liao E.C. (2017). Comparison of Allergenicity at Gly m 4 and Gly m Bd 30K of Soybean after Genetic Modification. J. Agric. Food Chem..

[26] Selb R., Wal J.M., Moreno F.J., Lovik M., Mills C., Hoffmann-Sommergruber K., Fernandez A. (2017). Assessment of endogenous allergenicity of genetically modified plants exemplified by soybean—Where do we stand?. Food Chem. Toxicol..

[27] Goodman R.E., Panda R., Ariyarathna H. (2013). Evaluation of Endogenous Allergens for the Safety Evaluation of Genetically Engineered Food Crops: Review of Potential Risks, Test Methods, Examples and Relevance. J. Agric. Food Chem..

[28] Graf L., Hayder H., Mueller U. (2014). Endogenous allergens in the regulatory assessment of genetically engineered crops. Food Chem. Toxicol..

[29] Fernandez A., Mills E.N., Lovik M., Spoek A., Germini A., Mikalsen A., Wal J.M. (2013). Endogenous allergens and compositional analysis in the allergenicity assessment of genetically modified plants. Food Chem. Toxicol..

[30] Panda R., Ariyarathna H., Amnuaycheewa P., Tetteh A., Pramod S.N., Taylor S.L., Ballmer-Weber B.K., Goodman R.E. (2013). Challenges in testing genetically modified crops for potential increases in endogenous allergen expression for safety. Allergy.

[31] EFSA Panel on Genetically Modified Organisms (GMO) (2017). Guidance on allergenicity assessment of genetically modified plants. EFSA J..

[32] Kyhse-Andersen J. (1984). Electroblotting of multiple gels: A simple apparatus without buffer tank for rapid transfer of proteins from polycrylamide to nitrocellulose. J. Biochem. Biophys. Methods.

[33] Hei W., Li Z., Ma X., He P. (2012). Determination of beta-conglycinin in soybean and soybean products using a sandwich enzyme-linked immunosorbent assay. Anal. Chim. Acta.

[34] Hanafusa K., Murakami H., Ueda T., Yano E., Zaima N., Moriyama T. (2018). Worm wounding increases levels of pollen-related food allergens in soybean (Glycine max). Biosci. Biotechnol. Biochem..

[35] Krishnan H.B., Kim W.S., Jang S., Kerley M.S. (2009). All three subunits of soybean beta-conglycinin are potential food allergens. J. Agric. Food Chem..

[36] Adachi A., Horikawa T., Shimizu H., Sarayama Y., Ogawa T., Sjolander S., Tanaka A., Moriyama T. (2009). Soybean beta-conglycinin as the main allergen in a patient with food-dependent exercise-induced anaphylaxis by tofu: Food processing alters pepsin resistance. J. Clin. Exp. Allergy.

[37] Natarajan S., Khan F., Song Q., Lakshman S., Cregan P., Scott R., Shipe E., Garrett W. (2016). Characterization of Soybean Storage and Allergen Proteins Affected by Environmental and Genetic Factors. J. Agric. Food Chem..

[38] Quirce S., Fernández-Nieto M., Polo F., Sastre J. (2002). Soybean trypsin inhibitor is an occupational inhalant allergen. J. Allergy Clin. Immunol..

[39] Amnuaycheewa P., Gonzalez de Mejia E. (2010). Purification, characterisation, and quantification of the soy allergen profilin (Gly m 3) in soy products. Food Chem..

